# Plant proteolytic enzyme papain abrogates angiogenic activation of human umbilical vein endothelial cells (HUVEC) *in vitro*

**DOI:** 10.1186/1472-6882-13-231

**Published:** 2013-09-21

**Authors:** Thomas Mohr, Lucia Desser

**Affiliations:** 1 ScienceConsult, Enzianweg 10a, A-2353, Guntramsdorf, Austria; 2 Marlyn Neutraceuticals, 4404 E Elwood, Phoenix, AZ 85040, USA

**Keywords:** Bromelain, Papain, Ficin, Angiogenesis, Endothelial cells, VEGF, Plant proteolytic enzymes

## Abstract

**Background:**

Vascular endothelial growth factor (VEGF) is a key regulator of physiologic and pathogenic angiogenesis in diseases such as cancer and diabetic retinopathy. It is known that cysteine proteases from plants, like bromelain and papain are capable to suppress inflammatory activation. Recent studies have demonstrated that they may interfere with angiogenesis related pathways as well. The aim of this study was to investigate the anti-angiogenic effects of papain on human umbilical vein endothelial cells (HUVEC) *in vitro*.

**Methods:**

Cell viability after prolonged treatment with papain was investigated by life cell staining and lactate dehydrogenase release assay. Angiogenic activation was assessed by ELISA against phosphorylated proteins AKT, MEK1/2, ERK1/2, SAPK/JNK and p38-MAPK. Growth inhibition was determined by means of an MTT-assay and cell migration by means of a scratch assay. Capability to form a capillary network was investigated using a tube formation assay.

**Results:**

Papain did not induce proteolysis or cell detachment of HUVEC in a concentration range between 0 and 25 μg/mL. Four hours treatment with 10 μg/mL papain resulted in a reduced susceptibility of endothelial cells to activation by VEGF as determined by phosphorylation levels of Akt, MEK1/2, SAPK/JNK. Papain exerted a distinct inhibitory effect on cell growth, cell migration and tube formation with inhibition of tube formation detectable at concentrations as low as 1 μg/mL. Bromelain and ficin displayed similar effects with regard to cell growth and tube formation.

**Conclusion:**

Papain showed a strong anti-angiogenic effect in VEGF activated HUVEC. This effect may be due to interference with AKT, MEK1/2 and SAPK/JNK phosphorylation. Two other plant derived cysteine proteases displayed similar inhibition of HUVEC cell growth and tube formation. These findings indicate that plant proteolytic enzymes may have potential as preventive and therapeutic agents against angiogenesis related human diseases.

## Background

Angiogenesis is the formation of new capillary blood vessels by a process of sprouting from pre-existing vessels and occurs during development as well as in a number of physiological and pathological settings. It plays a key role in chronic human diseases such as ocular disorders, rheumatoid arthritis and cancer [1]. In solid tumors, induction of angiogenesis is a necessary precondition for tumor growth beyond the size of a pinhead [2]. Mounting evidence suggests an important role for angiogenesis in hematologic malignancies as well [3]. In cardiovascular disease, the role of angiogenesis is much less clear. Angiogenic therapy is considered to be a promising approach to treat cardiovascular disease [4]. On the other hand, angiogenesis within the vessel wall is positively correlated with the development of atherosclerosis, because microvessels are abundant in atherosclerotic lesions and contribute significantly to disease progression and plaque instability [5,6].

Angiogenesis is mediated via a number of different agents, among them VEGF, bFGF, IGF and EGF and their receptors [7]. Upon binding of the ligands to the extracellular domain of their receptors, dimerization and autophosphorylation of the intracellular receptor tyrosine kinases occur, resulting in the activation of a cascade of downstream pathways including the PI3K pathway (cell survival), Src (vascular permeability), FAK, p38-MAPK and Smad 2/3 (cell migration) and PLCγ pathways (proliferation) [8].

Cysteine proteases from plants, namely pineapple, figs and papaya have been used in complementary medicine for decades and are currently under investigation in connection with diseases involving inflammation. The level of proof, method of enzyme administration and dose and quality of the studies vary, but beneficial effects were observed in a number of different models, including experimental allergic encephalomyelitis (EAE) model of the human autoimmune disease multiple sclerosis, carrageenan-induced pleurisy in the rat, immunologically mediated arteriosclerosis in rat aortic allografts, rheumatologic diseases in mice and humans, and allergic asthma (reviewed in [9] and [10]). Some studies demonstrated that bromelain had an efficacy similar to standard anti-inflammatory drugs such as dexamethasone [11,12] or non-steroidal anti-inflammatory agents (NSAIDs) [13-16]. However, the exact mode of action is unclear. Early studies suggested a modulation of the expression of surface molecules, particularly on immunocompetent cells [17] which likely interferes with cell-cell communication [18-20]. More recent studies found that cysteine proteases from plants prevent activation of inflammatory pathways such as the NF-κB, likely because of interference with the phosphorylation of IκBα [21-23]. Recently it has been demonstrated that Bromelain does not only inhibit NF-κB activation, but phosphorylation of AKT, ERK1/2, and p38-MAPK as well [21,22,24]. AKT, ERK1/2 and p-38 MAPK constitute important regulatory proteins in angiogenesis, regulating growth, migration and survival of endothelial cells [8]. These data suggest that plant derived cysteine proteases might not only display anti-inflammatory but an anti-angiogenic properties as well. We have therefore decided to investigate the effect of papain on angiogenic activation of HUVEC by VEGF. In this study we present to our knowledge for the first time data on the phosphorylation state of AKT, MEK1/2, ERK1/2, STAT3, SAPK/JNK and on key endothelial functions such as cell growth, migration and the capability to form tubes after treatment with papain. To put these data into a more general context, we tested whether other plant derived cysteine proteases display a similar effect on endothelial cell growth and tube formation.

## Methods

### Chemicals and media

Endothelial Basal Medium-2(MV) (Lonza, Verviers, Belgium) was supplemented with 10% FCS (Fetal calf serum, PAA, Pasching, Austria), hydrocortisone, ascorbic acid, gentamycin/amphotericin and growth factors bFGF, EGF, IGF, VEGF, according to the instructions of the manufacturer (EGM). EGM without growth factors and 2.5% serum supplement served as endothelial basal medium (EBM). Papain from papaya latex (lyophilized powder, chromatographically purified, cell culture grade, activity 10 U/mg protein) was purchased from Sigma-Aldrich, Vienna. Bromelain (lyophilized powder,chromatographically purified, activity 10 U/mg,) and ficin (lyophilized powder, chromatographically purified, activity 2 U/mg) were purchased from Sigma Aldrich, Vienna.

### Cell culture

HUVEC were purchased from Lonza (Verviers, Belgium) and cultured on tissue culture flasks coated with human fibronectin (Millipore, Vienna, Austria) in EGM containing 10% FCS. Upon reaching 80% confluence, cells were routinely passaged at a ratio of 1:2.

### Cell viability

Cells were harvested by trypsin detachment, seeded into fibronectin coated 12 well plates at a density of 120.000 cells per well and incubated with EGM to allow for the formation of monolayers. After 24 hours, EGM was replaced with EBM containing 2.5% FCS, 10 ng/mL VEGF (R&D Systems, Minneapolis, MN, USA), and 10 μg/mL papain (Sigma-Aldrich, Vienna, Austria). After a further 10 hours incubation period, cells were stained with 2 μM Calcein (Sigma-Aldrich, Vienna, Austria) and pictures were taken at 4 fold magnification using a Nikon Eclipse Ti, the FITC filter set of the instrument and a Nikon Digital Sight DS-Fi1C camera. Integrity of the monolayer and viability of cells was assessed visually.

### Lactate dehydrogenase (LDH) assays

Cells were seeded into 96-well microtiterplates at a density of 5000 cells per well and incubated over night under standard cell culture conditions in order to allow adherence of the cells and formation of monolayers. The next day medium was changed to EBM containing 2.5% FCS, 10 ng/mL VEGF and 10 respectively 25 μg/mL enzymes. Cells were incubated for another 24 hrs and released lactate dehydrogenase was determined using an 96-CytoTox Assay Kit (Promega, Heidelberg, Germany) according to the instructions of the manufacturer, including lysis of total cells to determine maximal LDH release.

Cell lysis was calculated according to

$$\mathrm{\%Lysis}=\frac{\left(OD_{\text{Sample}}-OD_{\text{untreated}}\right)}{\left(OD_{\text{max}}-OD_{\text{untreated}}\right)}\times 100$$

with OD_Sample_ being the optical density of the sample, Od_untreated_ being the optical density of the untreated control and Od_ma_ the optical density of a cell lysate obtained by treatement of cells with lysis buffer.

### Immunofluorescence staining

Cells were seeded into HFN coated 8-well chamber slides (Becton Dickinson, Franklin Lakes, NJ, USA), incubated for 24 hours in EGM to allow initial attachment and stimulated with 10 ng/mL LPS. Following treatment with 10 μg/mL papain, cells were fixed with 2% paraformaldehyde for 15' at RT and permeabilized with 0.1% Triton X-100 for 5' at RT. Cells were washed thrice with 300 μL PBS containing 1% FCS and incubated with primary antibody against VEGFR2 (mouse anti VEGFR2, Abcam, Cambridge, UK, dilution 1:200). After three further washes, cells were incubated with FITC labeled anti-mouse antibody (Sigma-Aldrich). Cells were washed for a final three times, enclosed in Vectrashield Hard-Set (Vector labs, Burlington, CA, USA) and scanned using a Zeiss LTM700 confocal laser microscope.

### Multitarget ELISA

Phosphorylated proteins were determined using multitarget ELISA kits (cell signaling technology, Beverly, MA, USA). Briefly, cells were seeded in HFN coated 25 cm^2^ tissue culture flasks and incubated in EGM. After 24 hours, EGM was changed to EBM. After a 24 hours starving period, medium was changed to EBM containing 10 μg/mL papain and cells were incubated for 4 hours. Cells were washed twice with PBS to remove papain and stimulated for 15 minutes with EBM containing 10 ng/mL VEGF. Protein isolation and determination was carried out according to the instructions of the manufacturer. Expression values were normalized to protein content of the sample and expressed as fold unstimulated control.

### MTT-assay

Cells were seeded into 96-well microtiterplates at a density of 2500 cells per well and incubated over night under standard cell culture conditions in order to allow adherence of the cells. The next day cells were treated as indicated (10 ng/mL VEGF respectively 10 ng/mL VEGF in combination with enzymes in EBM). Cells were incubated for another 72 hrs. Cell growth was determined using an EZ4U-assay (Biomedica, Vienna, Austria) according to the instructions of the manufacturer. Cells incubated in EBM without growth factors served as untreated control. Growth inhibition was calculated as fold control.

### Cell migration assay

Cells were detached by trypsin treatment and seeded into fibronectin coated 12-well microtiter plates at densities of 120.000 cells/well. After 24 hours incubation in EGM, the monolayer was scratched using a 200 μL pipette tip. Cells were stained with 1 μM calcein-AM (Sigma-Aldrich, Vienna) and photographed using the equipment as described above at 10 fold magnification. Medium was changed to EBM with 10 ng/mL VEGF, or 10 ng/mL VEGF and papain as indicated and cells were incubated for another 12 hrs. At the end of the incubation period, cells were again photographed as described above. The Tscratch software [25] was used to quantify the open area. Results were calculated as percent closure after 14 hours with the area at start being set to 100% and expressed as percent VEGF treated control.

### Tube formation assay

Angiogenesis slides (Ibi, Martinsried, Germany) were coated with 10 μL Matrigel per well (Growth Factor Reduced, Becton Dickinson) and incubated for 30 min at 37°C to allow for gelling. Endothelial cells were detached by trypsin treatment and centrifugated for 5' at 300 g. The pellet was washed once with PBS and adjusted to 100.000 cells per mL in EBM-2(MV) containing ascorbic acid and hydrocortisone according to the instructions of the manufacturer and 10 ng/mL VEGF. Cell suspension (50 μL per well) was seeded into each well. Cells were incubated to allow initial cell attachment. After 4 hours, enzymes were added as indicated and cells were further incubated. After 20 hours, cells were stained with 1 μM Calcein. Micrographs of fluorescent cells were taken as described above. Tube formation was quantified as described by Guidolin et al. [26] using the Cell Profiler Software Package [27]. Briefly, images were converted into binary images by thresholding as described by Otsu [28]. Areas with an extension of more than 125 μm in one direction were considered as tubes and selected for analysis, smaller areas were discarded. A single pixel topological skeleton representing the tubular network was constructed and network length was calculated by multiplying the pixel count with a scaling factor representing microns per pixel. All experiments were carried out in triplicates.

### Statistical analysis

Data are presented as mean ± standard error of measurement. Statistical analysis was carried out using GraphPad Prism. Differences were assessed by one-way ANOVA followed by Dunnett’s multiple comparison test.

## Results

### Cell viability

**Figure 1:**
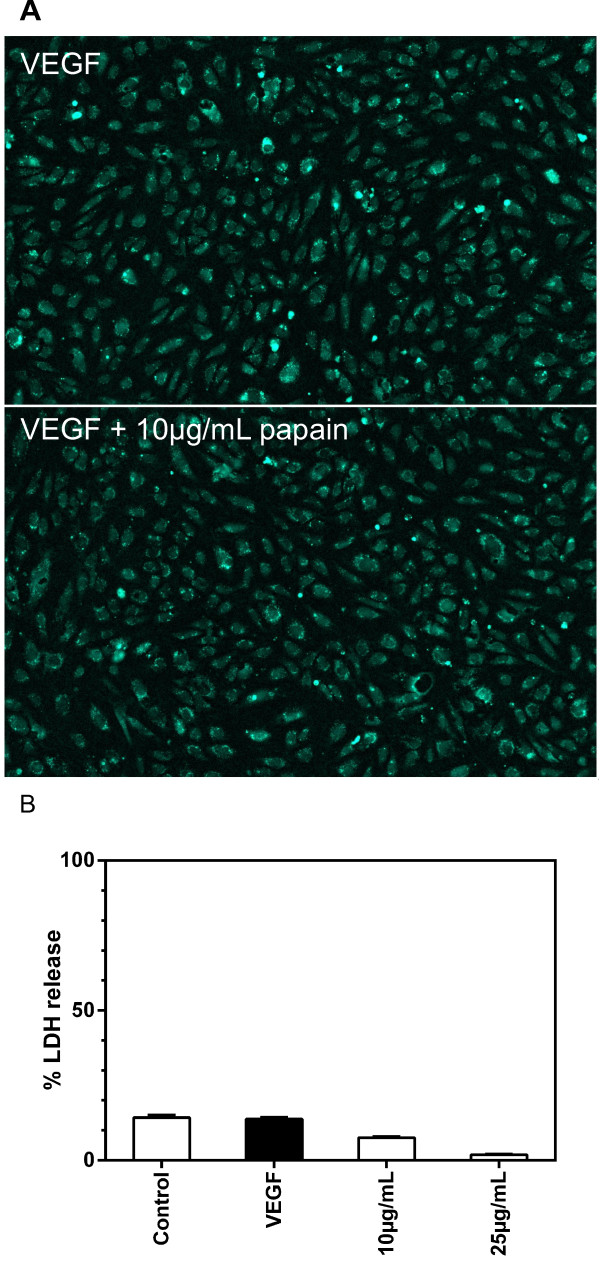
**Effect of papain on cell viability (panel A) and lactate dehydrogenase release (panel B).** To test cell viability, HUVEC were seeded into fibronectin coated microtiterplates and cultured in EGM. After 24 hours medium was changed to EBM and cells were cultured for 10 hours in EBM containing 10 ng/mL VEGF or 10 ng/mL VEGF and 10 μg/mL papain. Cells were stained using life cell staining calcein-AM. Cells were photographed at 4 fold magnification using a Nikon Eclipse Ti, the FITC filter set of the instrument and a Nikon Digital Sight DS-Fi1C CCD camera. Integrity of the monolayer and viability of cells was assessed visually. To investigate lactate dehydrogenase release, HUVEC were seeded into fibronectin coated 96-well microtiterplates and cultured in EGM. After 24 hours medium was changed to EBM containing 10 ng/mL VEGF or 10 ng/mL VEGF and papain at concentrations as indicated. After 48 hours, released lactate dehydrogenase was determined using a 96-Cytotoxassay kit. Data are shown as mean ± standard error of measurement. Monolayers of papain treated cells are intact and cells are viable as demonstrated by calcein uptake, metabolization and retention (panel **A**). No increase of LDH in the supernatant was apparent at enzyme concentrations of up to 25 μg/mL (panel **B**).

Proteases from various sources are routinely used in cell culture to detach cells from surfaces of tissue culture flasks. At higher concentrations and/or prolonged incubation time proteases are capable of damaging cells. To exclude this effects we first examined cell adhesion and cell viability after prolonged exposure to papain (Figure 1). After 10 hours incubation with 10 μg/mL papain cells are viable as demonstrated by calcein uptake, metabolization and retention. No detachment from the vessel surface was observed and cells retained a typical flat “cobblestone” morphology. These findings are confirmed by the lactate dehydrogenase (LDH) release assay which would be indicative for cell lysis (Figure 1B). No significant increase of LDH in the supernatant could be observed at concentrations of up to 25 μg/mL papain. These data demonstrate that papain does not damage cells under our test conditions, even under prolonged incubation times.

### Receptor integrity

**Figure 2:**
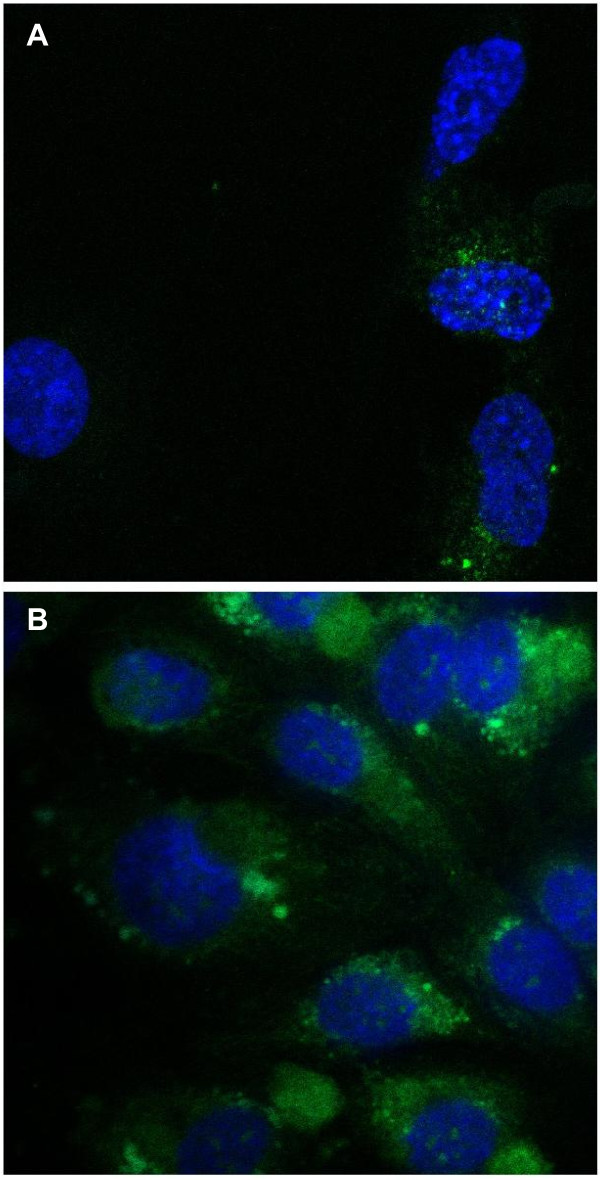
**Effect of papain on the expression of VEGF-receptor 2.** HUVEC were seeded into fibronectin coated 8-well chamber slides and cultured in EGM. After 24 hours, medium was changed and 10 ng/mL VEGF or a combination of 10 ng/mL VEGF and 10 μg/mL papain. After 1 hour, cells were fixed with 2% paraformaldehyde and stained with primary mouse antibody against VEGFR2 and FITC labeled goat anti mouse secondary antibody (Panel **B**). Cell nuclei were counter stained using DAPI. Unspecific mouse IgG served as isotype control (Panel **A**). Although there is slight unspecific binding of secondary antibody visible on the isotype control, VEGFR2 was clearly present on cells after treatment with 10 μg/mL papain.

Plant derived proteases are known to interfere with the expression of surface receptors of immunocompetent cells [17] therefore we investigated the expression of VEGF receptor 2 on HUVEC after 4 hour incubation with medium containing 10 μg/mL papain. HUVEC stained positive for VEGF receptor 2 (Figure 2) indicating that VEGF-receptor 2 is not shedded from the cell surface under our test conditions.

### Pathway activation

**Figure 3:**
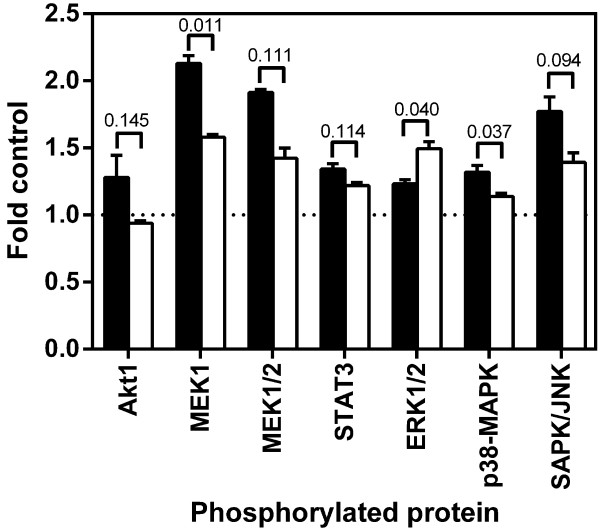
**Effect of pre-treatment of papain on the phosphorylation status of key-regulatory proteins.** HUVEC were seeded into fibronectin coated 25 cm^2^ tissue culture flasks and cultured in EGM. After 24 hours medium was changed to EBM containing 10 μg/mL Papain and cells were incubated for further four hours. Cells were washed with PBS to remove papain and treated for 15 minutes with EBM containing 10 ng/mL VEGF. Untreated cells served as control. Protein was isolated and phosphorylation was measured using a multipathway ELISA kit. Data are shown as mean ± standard error of measurement with the numbers indicating p-values, black bars indicating VEGF treatment alone and open bars indicating pre-treatment with papain. Pretreatment with papain profoundly interferes with the ability of cells to respond to angiogenic stimuli. Phosphorylation of MEK1 and p38-MAPK were significantly downregulated, whereas ERK1/2 was significantly upregulated. Phosphorylation of Akt1 and SAPK/JNK was also downregulated but with p-values between 0.1 and 0.15.

Next we investigated the effect of pre-incubation with papain on the phosphorylation status of key regulatory proteins AKT1, MEK, p38-MAPK, SAPK/JNK and STAT3 (Figure 3). Four hours pre-incubation with papain lead to a significantly reduced phosphorylation of MEK1 and p38-MAPK after activation with VEGF (2.13 vs 1.58 fold control resp. 1.32 to 1.14 fold control). Phosphorylation of AKT1 (1.28 vs. 0.94 fold control) and SAPK/JNK (1.77 vs 1.34 fold control) was reduced, although significance levels varied between 0.05 and 0.15. Surprisingly, ERK1/2 was significantly up-regulated (1.23 vs 1.50 fold control, p<0.05).

### Cell proliferation

**Figure 4:**
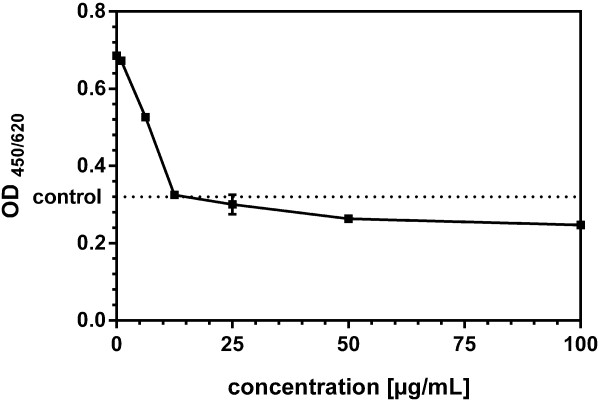
**Effect of papain on cell proliferation of HUVEC.** HUVEC were seeded into fibronectin coated 96-well microtiterplates and cultured in EGM. After 24 hours medium was changed to EBM containing 10 ng/mL VEGF or 10 ng/mL VEGF and papain at concentrations as indicated. After 72 hours, cell growth was assayed using an MTT-assay kit. Data are shown as optical density at 450 nm with a reference wavelength of 620 nm, mean ± standard error of measurement. Papain exerts a significant inhibition of proliferation at concentrations between 6.25 μg/mL and 100 μg/mL.

A switch from quiescence to proliferation is the first step in angiogenic activation. Whereas the anti-inflammatory action of plant proteases is well documented, much less is known about their effect on cell growth. We therefore investigated the effect of papain on the proliferation of VEGF stimulated endothelial cells (Figure 4). HUVEC were treated with 10 ng/mL VEGF with or without addition of papain at the indicated concentrations. Papain exerts a significant inhibition of proliferation at concentrations between 6.25 μg/mL and 100 μg/mL with an IC50 at 7.5 μg/mL Papain. At 100 μg/mL, metabolic activity as indicated by MTT metabolization was slightly below the untreated control. This is indicative for complete growth inhibition since endothelial cells in media lacking growth factors are not completely quiescent.

### Cell migration

**Figure 5:**
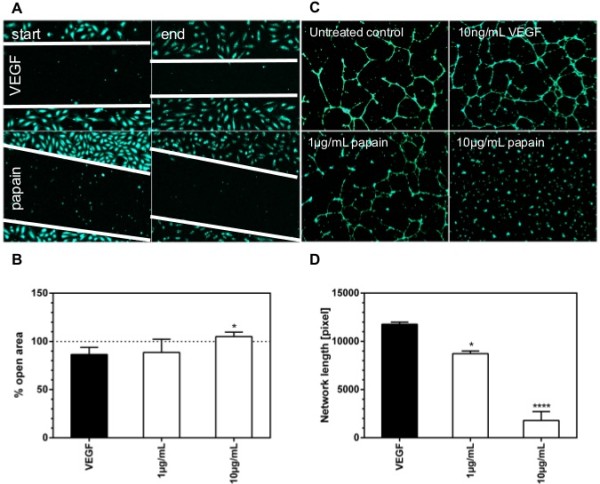
**Effect of proteolytic enzymes on cell migration and tube formation in HUVEC.** HUVEC were seeded into fibronectin coated 12-well microtiterplates and cultured in EGM. After 24 hours medium was changed to EBM. Cells were stained using calcein-AM, the monolayer was scratched with a 200 μL pipette tip and photographed at 10 fold magnification using a Nikon Eclipse Ti as described above. After 14 hours culture in EBM containing 10 ng/mL VEGF or 10 ng/mL VEGF and papain and photographed. covered area was measured using the TScratch software package, results were calculated as percent open area. Data are shown as mean ± SEM. Panel **A** shows photomicrographs at the beginning of the assay (column start) and after 14 hours incubation (column end) for cells treated with either 10 ng/mL VEGF or 10 ng/mL VEGF and 10 μg/mL papain. Migration fronts are marked by white lines. Panel **B** shows the percentage open area. Papain inhibited cell migration almost completely at a concentration of 10 μg/mL. Angiogenesis slides were coated with 10 μL Matrigel per well and incubated for 30 min at 37°C. Endothelial cells were and seeded into the wells at a density of 5000 cells per well in EBM containing 10 ng/mL VEGF. After 4 hours preincubation, papain was added at concentrations as indicated. After a further 20 hour incubation period, cells were labelled with 2 μM Calcein-AM. Micrographs of fluorescent cells were taken at 4 fold magnification using a Nikon Eclipse Ti as described above. Tube formation was quantified by the angiogenesis analyzer plugin for ImageJ. Panel **C** shows photomicrographs for the control, treatment with 10 ng/mL VEGF and with 10 ng/mL VEGF in combination with 1 and 10 μg/mL papain. Panel **D** shows the measured network length as percentage untreated control. Tube length decreased significantly to control levels after treatment with 1 μg/mL papain. At concentrations of 10 μg/mL tube formation was almost completely abrogated.

The next step in angiogenesis is migration of endothelial cells under influence of an angiogenic stimulus, therefore we investigated the effect of papain on cell migration (Figure 5A and B). HUVEC were treated with 10 ng/mL VEGF with or without addition of papain at the indicated concentrations. Papain showed a statistical significant inhibition of cell migration at a concentration of 10 μg/mL. At 1 μg/mL a slight inhibitory effect could be observed, however, it was not significant.

### Tube formation

To cover the final step of angiogenesis we investigated the effect of Papain on tube formation (Figure 5C and D). HUVEC were treated with 10 ng/mL VEGF with or without addition of papain at the indicated concentrations. VEGF significantly stimulated the formation of tubes. Network formation was inhibited significantly at a concentration of 1 μg/mL or papain. A concentration of 10 μg/mL abrogated tube formation almost completely.

### Proteases bromelain and ficin

**Figure 6:**
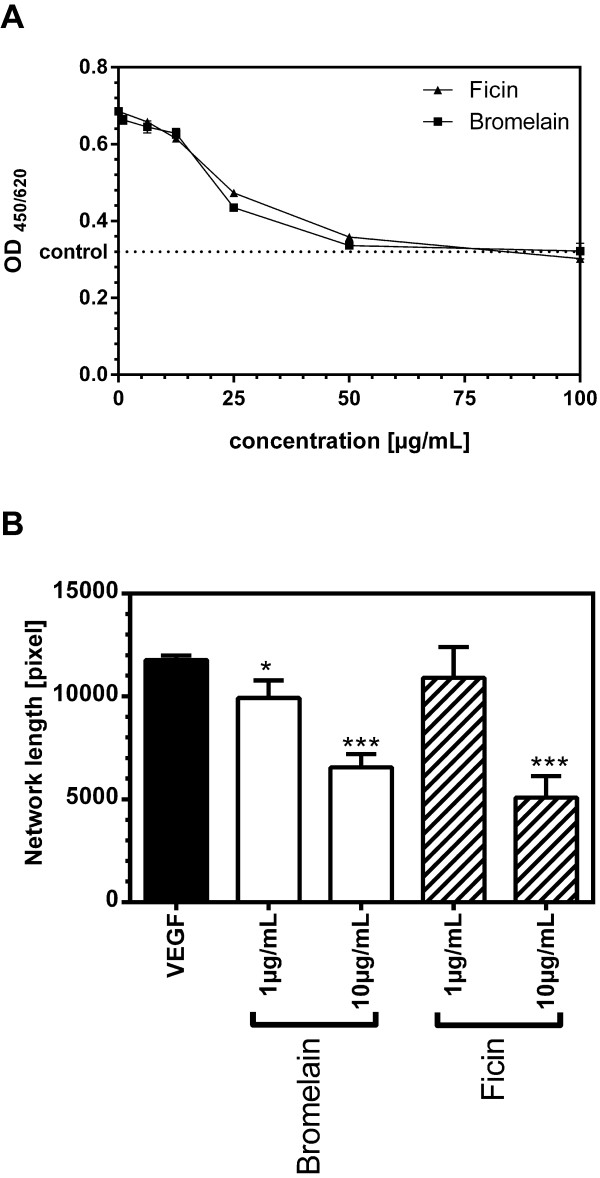
**Effect of other plant derived proteolytic enzymes on cell growth and tube formation of HUVEC.** HUVEC were seeded into fibronectin coated 96-well microtiterplates and cultured in EGM. After 24 hours medium was changed to EBM containing 10 ng/mL VEGF or 10 ng/mL VEGF and bromelain respectively ficin at concentrations as indicated. After 24 hours, cell growth was assayed as described above. Data are shown as mean ± standard error of measurement. Treatment with bromelain as well as ficin resulted in a significant inhibition of proliferation at concentrations between 10 μg/mL and 25 μg/mL (Panel **A**). Panel **B** shows the effect of bromelain and ficin on tube formation, assayed as described above. Briefly, after 4 hours preincubation bromelain respectively ficin was added at concentrations as indicated. Data were measured as network length and shown as mean percent control ± standard error of measurement. Treatment with 1μg/mL bromelain inhibited tube formation significantly to control levels. At concentrations of 10 μg/mL bromelain or ficin tube formation was almost completely abrogated.

Finally we investigated the capability of other plant derived cysteine proteases to inhibit endothelial cell growth (Figure 6A) and tube formation (Figure 6B). Bromelain as well as ficin inhibited growth of endothelial cells. We could determine the IC50 with 21.30 μg/mL for bromelain and 24.22 mg/mL for ficin. Both enzymes significantly inhibited tube formation at a concentration of 10 μg/mL. Bromelain exerted significant inhibition of tube formation already at a concentration of 1 μg/mL.

## Discussion

Plant derived cysteine proteases, especially bromelain have been used for decades in complimentary medicine as a remedy for inflammatory diseases. However, the mode of action by which plant cysteine proteases act is not well understood. Several mechanisms have been proposed: Cleavage of proteins from the cell surface [18], Interference with key components of pathways [21,22] and direct cytotoxicity via induction of apoptosis [23].

To address these questions we investigated direct cytotoxicity of papain towards endothelial cells. Prolonged incubation of endothelial cells with concentrations of up to 25 μg/mL papain did not result in increased release of lactate dehydrogenase which would be indicative for cell lysis. Additionally MTT assays showed that papain at concentrations of up to 100 μg/mL did suppress cell growth only to slightly below untreated control. These results are in line with other studies were protease concentrations of up to 400 μg/mL have been used [23]. Next we investigated the possibility of proteolytic removal of the main receptor for VEGF mediated angiogenesis, VEGFR2. Indeed, immunofluorescent staining showed that VEGFR2 remains on the cell surface even after prolonged incubation with papain.

We then investigated the phosphorylation status of AKT1, ERK1/2, MEK1/2, p38-MAPK and SAPK/JNK after pre-treatment with papain and found a modulation of phosphorylation levels of AKT1, MEK1/2, p38-MAPK and SAPK/JNK (down-regulated) and ERK1/2 (up-regulated). The PKB/AKT pathway is one of the main signaling pathways activated by VEGFR2 [8]. Down-regulation of AKT phosphorylation by cysteine proteases has been observed in other cells models in context with blocking NF-κB activation and subsequent induction of apoptosis [23]. However, in context of the VEGF receptor 2 signaling cascade, AKT phosphorylates BAD and caspase9 thus inhibiting their apoptotic activity. Additionally AKT phosphorylates the FOXO family of proteins which leads to their inactivation and a decrease in transcription of proteins promoting apoptosis [29]. In this context AKT acts as an inhibitor of apoptosis and is crucially required for VEGF mediated angiogenic activation [30-32]. Targeting AKT inhibits angiogenesis *in vivo*[33].

VEGF additionally signals via Ras → c-RAF → MEK → ERK [8]. An effect of cysteine proteases on this pathway has first been reported by Mynott et al. in a T-cell model [34]. In this model, treatment with bromelain down-regulated ERK-2 phosphorylation, and decreased IL-2, IFN-γ, and IL-4 mRNA accumulation. Since trypsin had no effect, Mynott could exclude unspecific proteolysis. Our data suggest that down-regulation of this signaling pathway includes down-regulation of MEK1/2 activity. MEK1/2 are protein kinases that mediate the phosphorylation of ERK1 or ERK2. This activates ERK1/2, which are protein-serine/threonine kinases with a broad spectrum of cytosolic and nuclear substrates. Interestingly we observed an up-regulation of ERK1/2 phosphorylation. This differs from the situation reported in other cell models and *in vivo* where a marked inhibition of ERK1/2 has been observed [21,34]. The reason for this observation may be the down-regulation of p38-MAPK which serves negative regulator for ERK1/2 [35].

Both p38-MAPK and JNK are mainly involved in cellular response to many types of stresses, but they also control proliferation, differentiation, survival and migration of specific cell types. Aside cellular stress JNK as well as p38 MAPK pathways can be activated by growth factors [36]. The role of SAPK/JNK in angiogenesis is not fully understood yet. Boyd et al. found that JNK inhibition lead to inhibition of tube formation [37] leading to the conclusion of JNK being a positive regulator of angiogenesis. This is underlined by the fact that inhibition of JNK significantly decreased endothelial proliferation and migration [38]. We observed a down-regulation of SAPK/JNK which cold in part explain the reduced migration and tube formation we observed.

Activation of p38-MAPK induces endothelial cell migration [39] and serves as a negative regulator for ERK1/2 and AKT in VEGF mediated angiogenesis [35]. On the other hand, in endothelial cells exposed to chronic inflammatory activation p38-MAPK acquires a pro-angiogenic role [40]. We observed a down-regulation of p38-MAPK which is in line with published observations in other models [21].

Finally we investigated whether these data translate into altered endothelial cell function (cell growth, cell migration and capability to form tubes). Papain inhibited cell growth in a concentration response dependent manner with an IC50 of 7 μg/mL. Cell migration was almost completely abrogated at a concentration of 10 μg/mL and tube formation was significantly inhibited at a concentration of 1 mg/mL. At a concentration of 10 μg/mL, tube formation was almost completely abrogated. Inhibition of cell growth and tube formation could also be seen in bromelain and ficin treated endothelial cells, pointing towards antiangiogenic properties of plant derived cysteine proteases in general.

## Conclusion

Papain displayed a strong anti-angiogenic effect in VEGF activated HUVEC which could also be seen with bromelain and ficin. This effect is likely caused by interference with key signaling pathways AKT, MEK, ERK1/2, p38-MAPK and SAPK/JNK signaling. These findings indicate that plant proteolytic enzymes effectively interfere with angiogenesis and that these proteases may have potential as preventive and therapeutic agents in diseases involving pathological angiogenesis.

## Competing interests

Both authors hold patents on the reduction of angiogenesis by plant proteolytic enzymes. Author LD is an employee of the financier of the study, Marlyn Neutraceuticals, Phoenix, Arizona. Her involvement encompassed conceiving the study, involvement with the designing of the study and help with data evaluation and manuscript correction.

## Author’s contributions

TM was conceived and designed the study, carried out the experiments, evaluated the data, interpreted the results and wrote the manuscript. LD conceived the study, and helped with design, data evaluation and correcting the manuscript. Both authors read and approved the final manuscript.

## Pre-publication history

The pre-publication history for this paper can be accessed here:

http://www.biomedcentral.com/1472-6882/13/231/prepub
